# PepFun: Open Source Protocols for Peptide-Related Computational Analysis

**DOI:** 10.3390/molecules26061664

**Published:** 2021-03-16

**Authors:** Rodrigo Ochoa, Pilar Cossio

**Affiliations:** 1 Biophysics of Tropical Diseases, Max Planck Tandem Group, University of Antioquia, Medellin 050010, Colombia; rodrigo.ochoa@udea.edu.co; 2 Department of Theoretical Biophysics, Max Planck Institute of Biophysics, 60348 Frankfurt am Main, Germany

**Keywords:** peptide, python, bioinformatics, cheminformatics

## Abstract

Peptide research has increased during the last years due to their applications as biomarkers, therapeutic alternatives or as antigenic sub-units in vaccines. The implementation of computational resources have facilitated the identification of novel sequences, the prediction of properties, and the modelling of structures. However, there is still a lack of open source protocols that enable their straightforward analysis. Here, we present PepFun, a compilation of bioinformatics and cheminformatics functionalities that are easy to implement and customize for studying peptides at different levels: sequence, structure and their interactions with proteins. PepFun enables calculating multiple characteristics for massive sets of peptide sequences, and obtaining different structural observables derived from protein-peptide complexes. In addition, random or guided library design of peptide sequences can be customized for screening campaigns. The package has been created under the python language based on built-in functions and methods available in the open source projects BioPython and RDKit. We present two tutorials where we tested peptide binders of the MHC class II and the Granzyme B protease.

## 1. Introduction

The study and application of peptides is nowadays an active field for different research areas, including drug discovery [1]. Specifically, the development of synthetic peptides provides a novel route to disease treatment by overcoming some problems encountered, for example, with small molecules, such as low specificity during the binding processes and the generation of adverse effects caused by synthetic chemical groups [2,3]. Other applications include the identification of peptide-based biomarkers for diagnosis [4], or their roles as antigenic sub-units vaccine development [5]. However, the use of peptides has some limitations that include poor chemical and physical stability, short circulating plasma half-life, and solubility issues [3]. This motivates the analysis of peptides in silico using tools able to predict physico-chemical properties, as well as model and simulate their interactions with other molecules [6].

There is a diverse set of computational protocols for studying amino acid sequences and predicting the physico-chemical characteristics [7,8]. For example, the amino acid sequence of oligopeptides can be analyzed using bioinformatics tools designed to study proteins [9,10]. However, certain modifications or assumptions should be made to decrease the false positive rate or incorrect property predictions in the context of peptides. Evolutionary algorithms, which have been customized to align proteins based on generating gaps to detect potential homologues, do not align well massive peptide-sequence sets if the peptides are considered as ligands. To avoid this issue, one alternative is using position-by-position alignments weighted based on available position-specific scoring matrices [11]. In addition, some properties, such as empirical rules related with potential solubility and synthesis issues, are characteristic of peptides.

Descriptors based on sequence features have been extracted using machine learning protocols, classifying peptides according to particular characteristics [12]. These methods have been successful for predicting antimicrobial or anticancer peptide sequences as potential therapeutics [13]. In this scenario, large datasets of sequences provide a background for learning the associated properties of peptides. However, training data might not always be available. Moreover, the structural and dynamical information of the peptides is usually required to have a better understanding of their detail molecular mechanisms [14].

Characterizing a peptide’s most probable 3D structure is important to assess how it performs its biological function and interacts with proteins or small molecules [15]. Some structures can be obtained from the Protein Data Bank (PDB) [16]. These coordinates can be used as templates to predict the bound conformations of other peptide complexes using docking-based protocols [17] or modelling approaches that are mostly available through public web servers [18,19]. However, a challenge is still to predict accurate 3D models for large sets of peptides.

Peptides are composed of amino acids (as are proteins) but they are commonly studied as ligands. Therefore, the available tools for characterizing peptides are typically divided in two separate sets: those for studying proteins or those for studying small molecules. Standard repositories (or web servers) perform just one or two tasks, specialized in the prediction of a particular characteristic [20,21]. This motivates the implementation of an open source tool to generate easy-to-follow pipelines for developers and users that integrates diverse functionalities from both the protein or small molecule perspectives. Here, we present PepFun, an easy-to-use set of python functions personalized for the study of peptides. Using as input sequences or structural information, the user can obtain descriptive and predictive information about peptides of different sizes, and use auxiliary functions to create chemically-diverse peptide libraries. The code is open source, and it uses BioPython for embedding common bioinformatics protocols [22], and RDKit (https://www.rdkit.org/ accessed on 12 March 2021) for analyzing chemical entities. The available of validated and published tools to predict peptide properties allowed us to compile PepFun as an open repository to run, in a centralized manner, calculations on massive sets of peptides. In the following, we describe PepFun, and we provide examples for performing some peptide sequence- and structure-based analysis. Finally, we explain additional PepFun tools for creating diverse peptide libraries.

## 2. Results and Discussion

### 2.1. The PepFun Code and Functionalities

**Figure 1:**
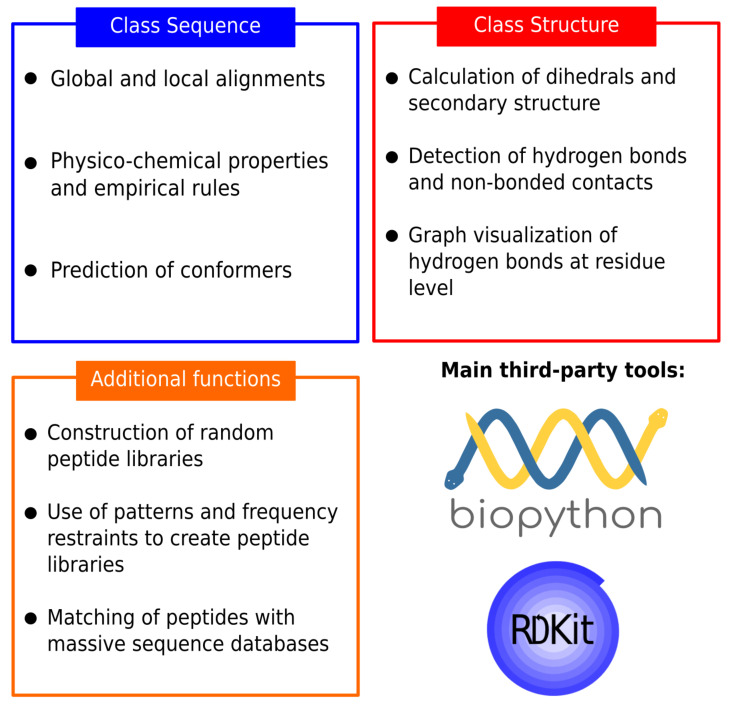
Summary of the PepFun modules. The modules are split into three categories: sequence-based tools, structure-based tools and additional functions. These modules rely on the Biopython functions for bioinformatics tasks, and the RDKit package for cheminformatics calculations.

PepFun is a compilation of bioinformatics and chemoinformatics functionalities that are easy to implement and personalize for studying peptides at different levels: sequence, structure and their interactions. The functions are part of an stand-alone tool that can predict multiple peptide characteristics, align their sequences with customized protocols, predict conformers, and use structural information to describe their secondary structure and analyze interactions with protein targets. More details are explained in the Methods. The scripts have been designed to be used by beginners in the field, or developers who want to embed the PepFun functionalities into more elaborated pipelines. A summary of the PepFun modules and functions is shown in Figure 1.

### 2.2. Installing and Running PepFun

The PepFun code can be downloaded from https://github.com/rochoa85/pepfun accessed on 12 March 2021. All the classes and functions of PepFun were written in python3, using built-in modules and functions provided by the BioPython and RDKit packages. These third-party tools can be installed using the available source codes, or through Conda (https://docs.conda.io/projects/conda accessed on 13 January 2021) virtual enviroments created with all the required packages embedded within the python code. The project was built and tested using the Travis CI framework (https://travis-ci.org/ accessed on 13 January 2021), automatizing the setup of the dependencies, the required validations and tests on different operating systems and virtual environments. A jupyter notebook is also available with a full step-by-step tutorial to run single and massive-set analysis with PepFun modules.

### 2.3. PepFun Tutorial and Examples

**Figure 2:**
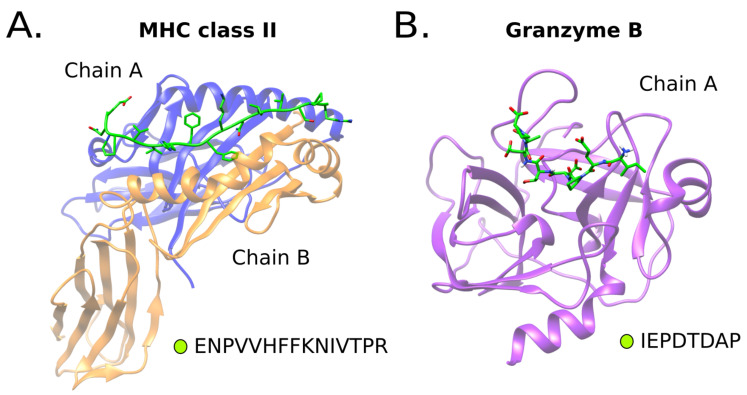
Protein peptide benchmark systems. Two systems were included in the analysis. An MHC class II allele structure (PDB id 1t5x) bound to a 15-mer peptide (**A**), and a Granzyme B protease protein (PDB id 1iau) bound to a 8-mer peptide substrate (**B**).

We used two benchmark systems of peptides binders, the Major Histocompatibility Complex (MHC) class II and Granzyme B protease, for comparing the predictions and results of PepFun. Graphical representations of the structures of these systems are shown in Figure 2. Both systems have available a diverse set of peptide binders that were used as reference to test some functions. The examples shown here are part of the tutorials provided within the source code.

#### 2.3.1. Analysis of Sequence Properties

**Figure 3:**
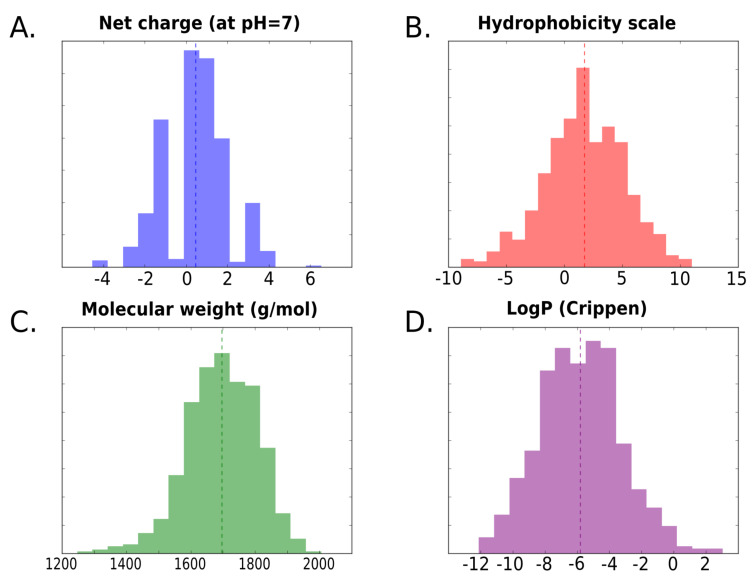
Distribution of peptide properties calculated with sequence-based functions of PepFun. For the 655 peptides reported as binders of the MHC class II allele, four properties were calculated: the net charge at pH = 7 (**A**), hydrophobicty with the Eisenberg scale [23] (**B**), the molecular weight (g/mol) (**C**) and the Crippen LogP [24] (**D**).

The PepFun functions for sequence analysis enable the prediction of multiple properties using bioinformatics and cheminformatics tools. The user only requires the peptide sequence, which can be read by PepFun using the command:
python pepfun.py - m sequence - s [SEQUENCE]
where the [SEQUENCE] field will be the amino acid sequence of the peptide. To test some of the sequence-based functionalities, the calculation of several peptide physico-chemical properties was ran using the datasets chosen from the benchmark systems (i.e., the MHC class II and the Granzyme B protease; see the Methods). For each set of peptide binders, we calculated the distribution of four properties: the net charge, hydrophobicity score [23], logP values [24] and molecular weight. In Figure 3, we show the distribution of the values for the MHC class II set of sequence binders.

The goal is to demonstrate how PepFun can be implemented to analyze libraries of peptides for discovering characteristics of their components, such as the identification of a dominant property, or by contrast, realize how promiscuous is the system to bind peptides with different characteristics. The average values from Table 1 are included to describe general trends of the observables in the libraries, but detailed analyzes from the distributions (e.g., presented in Figure 3) are recommended. For example, we found, for the MHC class II binders, that the peptides have a wide range of values for the predicted properties, including hydrophobic to hydrophilic sequences, which is characteristic of the receptor’s promiscuity [25].

A similar analysis was performed for a dataset of protease substrates, the results are shown in Supplementary Figure S1. On average the protease-binding peptides tend to be negatively charged, are smaller than the MHC peptide binders, and less hydrophobic. However, based on a study of proteases specificity profiles [27], the sequences can be diverse in terms of their physico-chemical properties such as the hydrophobicity and net charge. Therefore, challenges remain in the prediction of substrate cleavage patterns by machine learning and sequence-based methodologies, which can be aided by tools like PepFun.

In general, the methods provided in PepFun can automatize the calculation of properties of larger peptide-sequence libraries in short computational times, allowing for the construction of improved sequence sets based on desired properties and average trends. Another advantage of PepFun is the possibility to combine properties calculated from the amino acid sequence, with those obtained from the chemical SMILES representation of the molecule. An example of the properties calculated for the peptide binders of both selected systems, using as input amino acid sequences and SMILES representations is provided in Table 1. The table contains the average properties from the sets of peptides.

If the PepFun code is accessed from an external module, it is possible to run different types of pair-wise alignments (using peptide sequences as input). For example, tools for aligning peptides position-by-position that use different types of penalization for each match, or mismatch, are included [28]. In addition, a function to run blastp [29] online from BioPython is available, with its parameters adjusted for peptide comparisons. This is helpful to guide the finding of similar peptides in large databases. Examples of how to run these alignments are available in a Jupyter tutorial provided in the code repository.

#### 2.3.2. Structural Analysis of Peptides in Complex with Protein Targets

With PepFun it is possible to analyze a structure of an isolated peptide, or a peptide interacting with a protein. The PepFun functions enable the calculation of different observables, including the secondary structure or contact-interactions formed with the protein. For a structure of a complex, with PepFun one can count, for example, the number of non-bonded contacts based on a defined distance threshold. An example of how to run this kind of analysis is:
python pepfun.py - m structure - p [STRUCTURE_FILE] - c [CHAIN] - t [THRESHOLD]
where [STRUCTURE_FILE] is the protein-peptide complex, [CHAIN] is the chain id of the peptide and [THRESHOLD] is the distance to define a contact between the peptide and the protein target in Å. To test these capabilities, we calculated the average number of non-bonded contacts created by each position of an 8-mer peptide bound to the protease of reference (see Table 2). P1 position is the amino acid in the peptide that is cleaved by the enzyme’s catalytic residues, which is usually buried inside the protease’s active site, and has a large number of contacts [30]. In contrary, the flanking amino acids (see Figure 2B) of the peptide tend to have less contacts, mostly because this is the part of the peptide that detaches after the cleavage reaction happens [30]. This analysis can be related to the intrinsic promiscuity of the protease’s binding region [31]. In ref. [27], we found that several structural descriptors, which are normally not used for cleavage predictions (because of the lack of protease structures bound to complete substrates), can provide relevant insights about the enzyme specificity.

**Figure 4:**
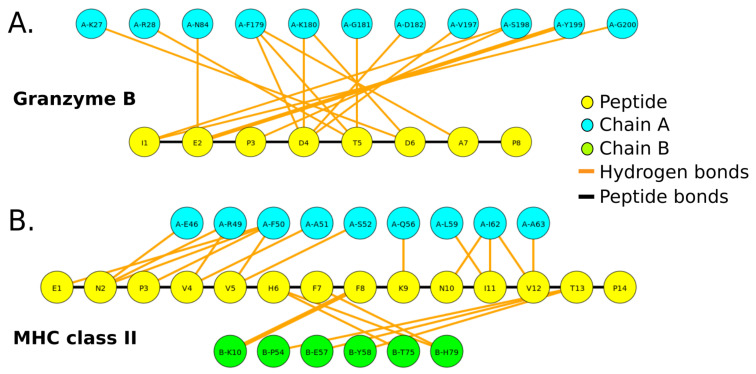
Visualization of potential hydrogen bonds between a peptide and residues of the protein binding site. The graphs were generated for the protease bound to an 8-mer peptide substrate (**A**), and MHC class II allele bound to a 15-mer peptide (**B**). The yellow nodes represent the peptide amino acids, and the other nodes are the residues of the protein interacting with the peptide (colored by chain). The bonds are represented by orange lines. The width of the lines is proportional to the number of hydrogen bonds between the pair of residues.

The second type of interactions calculated with PepFun are the potential hydrogen bonds. These can be counted but also represented graphically using a graph-based representation of the hydrogen bonds, where the amino acids are shown as nodes of different colors (depending on the chain). The hydrogen bonds are shown as lines, whose width depends on the number of bonds between the residue-pair. Examples of the graphical representations of the protein-peptide hydrogen bonds for the benchmark protein-peptide complexes are shown in Figure 4. Due to the chemical complexity and higher number of atoms involved in protein-peptide interfaces calculated with software like LigPlot+ [32], it is useful to obtain a simple schematic representation of the interactions at the residue level, which can be automatically generated with PepFun using the structure of the complex. For running this functionality, it is necessary to define the chain id that identifies the peptide, and also select if the peptide is linear or cyclic (which modifies the output to a Fruchterman-Reingold layout [33]).

#### 2.3.3. Peptide Libraries

Some additional functionalities of PepFun include the options to generate peptide libraries based on required patterns or amino acid frequencies. This is useful, not only for people working in virtual screening of peptides against a molecular target, but also for experimentalists that require the design of peptide libraries following rules and diversity criteria. As an example, we constructed two kinds of libraries from scratch. The first generates a uniform representation of each natural amino acid in all the positions of the peptide. The second generates all possible sequences following the pattern “XRTEX”, where X can be any of the natural amino acids uniformly distributed. A logo representation of each library [34] are shown in Supplementary Figure S2. The logos for the libraries of peptide binders from the benchmark systems are also shown. The libraries can be created also with D-amino acids using the HELM nomenclature to represent non-natural amino acids [35].

## 3. Materials and Methods

### 3.1. PepFun Technical Considerations

The Pepfun functionalities were originally designed under the Ubuntu 16.04 operating system. However, the project can be installed in any Conda virtual environment with the required dependencies, i.e., the third-party tools to run the bioinformatics and cheminformatics analysis such as Biopython and RDKit. PepFun can be used under other operating systems with the corresponding paths provided. A guide to run different examples is available in the code repository https://github.com/rochoa85/pepfun accessed on 12 March 2021.

### 3.2. PepFun Functionalities

#### 3.2.1. Sequence-Based Functionalities

This section is split into three main categories: alignments, properties and a conformer prediction. The alignments involve the implementation of position-specific scoring matrices to perform position-by-position matches between the query and subject peptides [28]. In addition, an online blastp function is provided with parameters optimized for aligning peptides against massive databases [36]. These are different from the common parameters used for protein-sequence alignment, which rely on opening and scoring gaps associated to evolution events, which are not required for peptide-based analysis.

The peptide properties are calculated using bio- and chem-informatics strategies that have been tested and validated extensively in the past. Specifically, the amino acid sequence is used to obtain information from reported amino acid parameters, including hydrophobicity [23], charges, and properties from the ProtParam project such as the aromaticity, instability index and isoelectric point [9]. The amino acid sequence can also be used to calculate empirical rules associated to the peptide’s synthesis and solubility viability. The identification of certain patterns within the peptide sequence can suggest if it could restrict experimental analysis [26]. The larger the number of rules violated, the lower the probability to be successfully synthesized and solubilized. Examples of such rules are if the number of charged and/or of hydrophobic amino acids exceeds 45% of the sequence, or if the absolute total peptide charge at pH 7 is more than 1, then it is probably not possible to synthesize it. The full list of rules are detailed within the code README file and the generated reports. Finally, the SMILES representation of the peptide is used as reference to calculate a number of properties available from the RDKit package, including the number of hydrogen donors and acceptors, the molecular weight and the Crippen logP coefficient [24], which is an estimation of the octanol/water partition coefficient using the Ghose/Crippen approach available in the RDKit project.

With the sequence information it is also possible to predict a conformer of the peptide using protocols available in RDKit. Specifically, the peptide SMILES is used as input, which is generated following a standard convention of the atoms’ numeration, enabling the creation of a PDB file with the residues numbered and ordered according to their peptide bonds [37]. The method used in RDKit to predict the conformer is the distance geometry approach [38]. It consists of calculating a distance bounds matrix that is smoothed using a triangle-bounds smoothing algorithm. Then, a random distance matrix that satisfies the bounds matrix is generated. The distance matrix is embedded in three-dimensions, producing the corresponding coordinates that are cleaned up using force fields such as the Merck Molecular Force Field (MMFF94) [39].

#### 3.2.2. Structure-Based Functionalities

Given the availability of peptide and protein-peptide complex structures (e.g., from the PDB), a set of PepFun functions were designed to analyze their properties and interactions. PepFun uses the DSSP package v3 to extract the secondary structure elements, as well as the calculation of the relative accessible solvent area for each residue in the peptide [40].

The analysis of the interactions involves the calculation of potential hydrogen bonds and non-bonded contacts between the peptide and the protein across the interface. The potential hydrogen bonds are calculated with DSSP, and a visualization of the interaction is generated using the igraph module of python [41]. Specifically, the peptide and the protein residues that are interacting are represented by nodes, and the potential hydrogen bonds are represented by lines and their width depends on the number of hydrogen bonds detected per pair of residues. The graph layout can change depending if the peptide is linear or cyclic. The non-bonded contacts are calculated using Biopython modules able to detect all the amino acid atoms interacting using the distances among the atoms. A threshold must be provided to define a contact. Typically, a threshold of 4.0 is used.

#### 3.2.3. Functions for Customizing Peptide Libraries

In addition to the classes designed for running sequence or structure-based functionalities, a set of functions are available to generate and analyze the content from peptide libraries. Libraries -from scratch- can be constructed following uniform distributions of the amino acids, or based on patterns required in the sequences. Combinatorial modules available in python are useful to quickly generate the population of sequences, which include the use of non-natural amino acids (i.e., D-amino acids), as an attempt for future versions to study peptidomimetics.

### 3.3. Test of PepFun with Sets of Known Peptide Binders

To test the implementation of PepFun, two well-known protein-peptide system with available sets of peptide binders were used. One involves the Major Histocompatibility Complex (MHC) class II, which has a large dataset of peptide binders available for different alleles [42]. A set of peptides with bioactivity data $\left(IC_{50}<50\mathrm{nM}\right)$ was chosen to analyze the distribution of multiple properties within the dataset [43]. The library has 655 peptides composed of 15 amino acids in length. The peptide structures were modelled in complex with the MHC class II allele DRB1*0101, with PDB id 1t5x. The modelling consisted on generating the new sequence by iterative single substitutions of the peptide template. The mutations were performed using the package fixbb from Rosetta [44], which was chosen based on a previous benchmark of other available mutation protocols [45]. After each substitution, the most probable rotamer from a dictionary of backbone-dependent conformations is selected, and the side chain atoms are relaxed with the backbone fixed.

The second system is a serine protease, granzyme B, which has available data of physiologically active substrates [46], stored in the MEROPS database [47]. A total of 599 peptides of 8 amino acids were selected. All the peptides were modelled using the structure with PDB id 1iau as reference, based on the methodology explained for the previous system.

## 4. Conclusions

**Figure 5:**
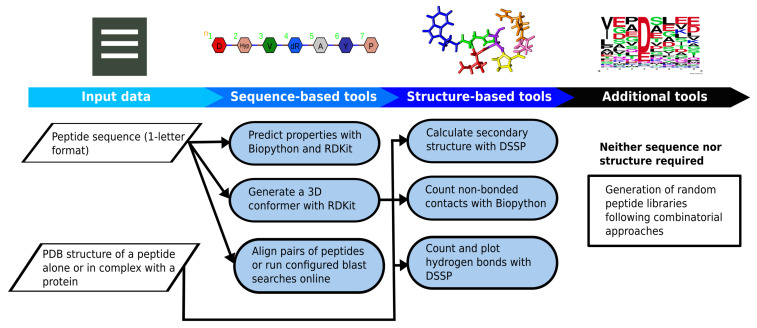
Flowchart of the PepFun repository based on the sequence, structure and additional tools provided to analyze massive datasets of peptides.

The PepFun package provides a set of functions suitable to perform bioinformatics and cheminformatics analysis over peptide sequences and structures, with the integration of easy-to-install dependencies using the python scripting language and a Conda virtual environment. The open source modules and classes enable the calculation of peptide properties, alignments of sequences, and the study of structural interactions, among other common tasks made by users working in the field. A flowchart of the PepFun code and main functionalities is provided in Figure 5. All-in-all, the method can be implemented by users with no programming expertise, or by developers able to embed the functions in complex bioinformatics pipelines dedicated to analyze peptides and their biological roles.

## Figures and Tables

**Table 1:** Average values of properties calculated with PepFun for the datasets of peptides associated to the MHC class II protein and the Granzyme B protease. We report the average net charge based on pka values of each amino acid at pH 7, the molecular weight calculated in g/mol using the SMILES representation of the peptide, the Crippen estimation of the octanol/water partition coefficient (logP) available in the RDKit [24], the average hydrophobicity from the Eisenberg scale [23], the isoelectric point and aromaticity obtained from the ProtParam package [9], the instability index from ProtParam as an estimate of the stability of the peptide in a test tube [9], the number of hydrogen bond acceptors and donors calculated using the SMILES representation, and the number of failed solubility and synthesis empirical rules [26]. We note that the higher the number of rules violated, the lower the probability to be solubilized or synthesized experimentally.

Property	Average Values MHC Set	Average Values Protease Set
Net charge	0.457	−1.872
Molecular weight (g/mol)	1696.111	884.133
LogP	−5.810	−4.235
Hydrophobicity	1.735	0.002
Aromaticity	0.099	0.043
Instability index	33.883	37.095
Isoelectric point	7.290	4.375
Number hydrogen donors	24.183	13.337
Number hydrogen acceptors	23.670	13.578
Number of solubility rules failed	2	1.5
Number of synthesis rules failed	4.5	0.5

**Table 2:** Average and standard deviation of the number of non-bonded contacts between each position of the 8-mer peptide and the protein. The positions are named according to the standard nomenclature defined for proteases binding sites [30].

Peptide Position	Average Number of Contacts
P4	13.64 ± 6.57
P3	12.63 ± 1.66
P2	18.70 ± 12.37
P1	45.59 ± 4.90
P1’	15.25 ± 8.68
P2’	7.89 ± 6.71
P3’	2.41 ± 1.85
P4’	0.02 ± 0.19

## Data Availability

The PepFun code is open-source and available at: https://github.com/rochoa85/PepFun accessed on 12 March 2021.

## Supplementary Materials

The following are available online, Figure S1: Distributions of peptide properties calculated with the sequence-based functions of PepFun, Figure S2: Logo of the libraries generated and used to test the PepFun functionalities.
