# Supplementary material for: PepFun: Open Source Protocols for Peptide-Related Computational Analysis
Source: Molecules. 2021 Mar 16;26(6):1664. doi: 10.3390/molecules26061664 (PMC8002403; doi:10.3390/molecules26061664)
Supplement: Supplementary file 1 [file molecules-26-01664-s001.pdf]

Supplementary Information

PepFun: open source protocols for peptide-related computational analysis

Rodrigo Ochoa[1] and Pilar Cossio[1,2]

[1]Biophysics of Tropical Diseases, Max Planck Tandem Group, University of Antioquia, 050010 Medellín, Colombia.

[2]Department of Theoretical Biophysics, Max Planck Institute of Biophysics, 60438 Frankfurt am Main, Germany.

Supplementary Figures

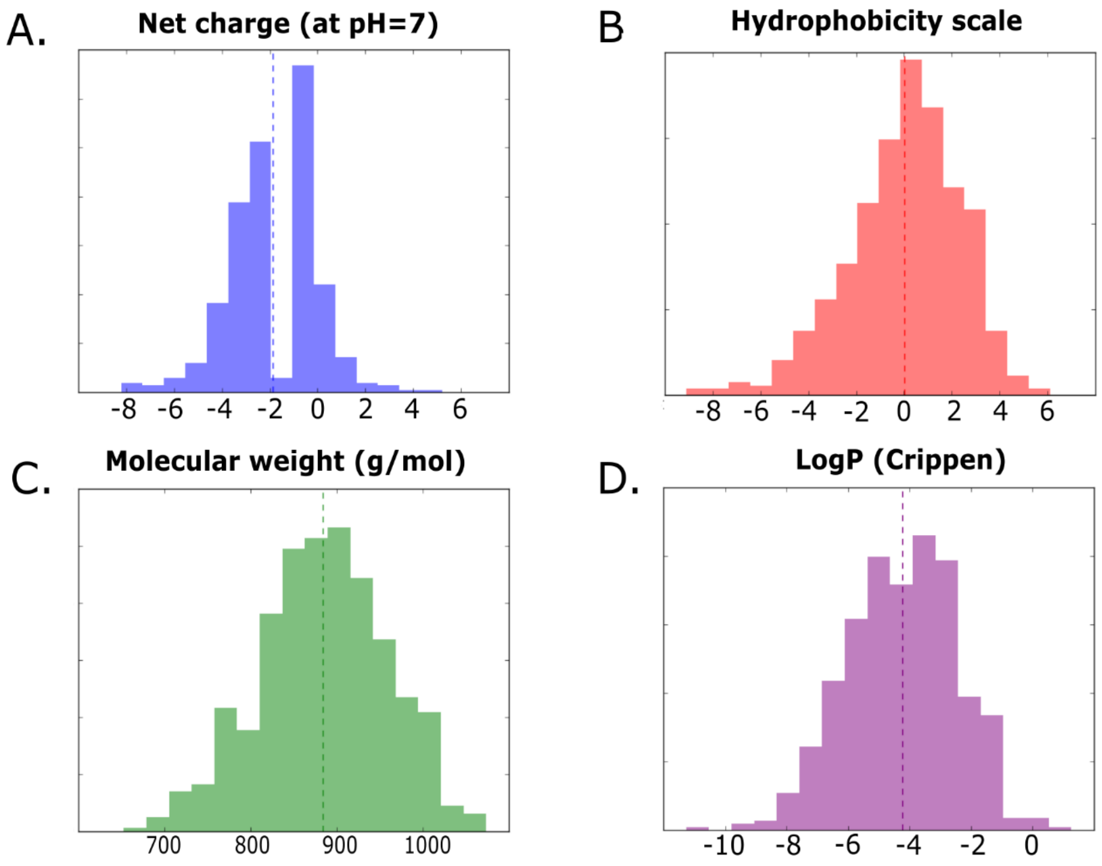

Figure S1. Distributions of peptide properties calculated with the sequence-based functions of PepFun. For the 599 peptides reported as substrates of the Granzyme B protease, four properties were calculated: the net charge (A), hydrophobicity with the Eisenberg scale [1] (B), the molecular weight (C) and the Crippen LogP [2] (D).

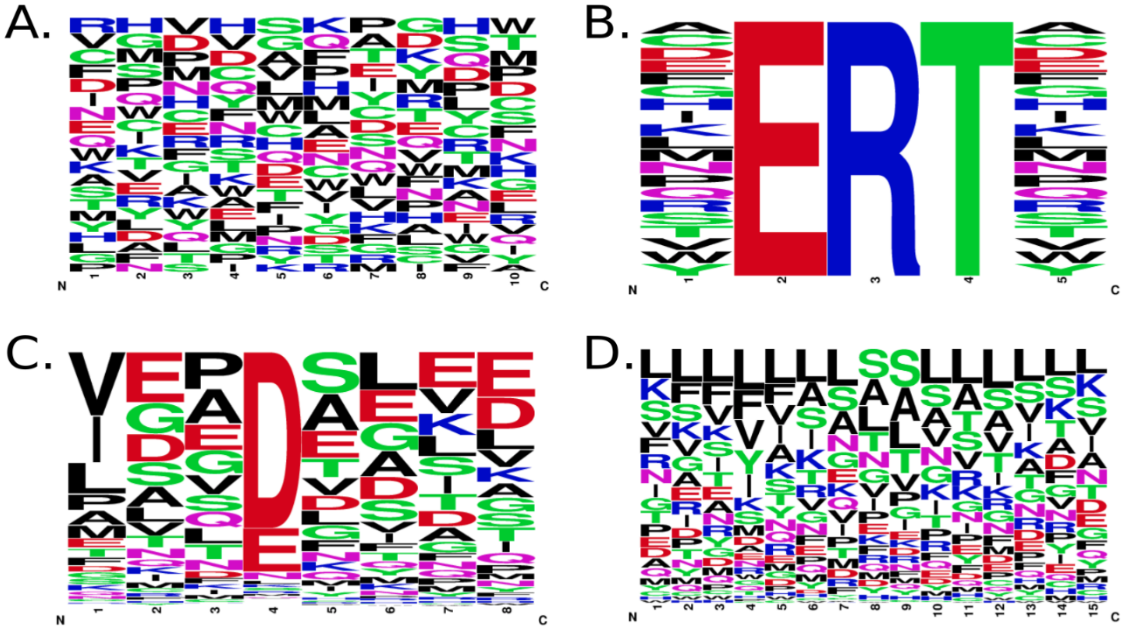

Figure S2. Logo of the libraries generated and used to test the PepFun functionalities. (A) Library of equally distributed amino acids in peptides of 10 amino acids long. (B) Library of 5-mer peptides using the pattern XERTX. (C) Library of known peptide substrates of the serine protease granzyme B. (D) Library of 15-mer peptides with bioactivity data against the MHC class II allele DRB1:0101. The last two come from experimental datasets.

References

[1] Eisenberg, D., Weiss, R. M., & Terwilliger, T. C. (1984). The hydrophobic moment detects periodicity in protein hydrophobicity. *Proceedings of the National Academy of Sciences*, *81*(1), 140-144.

[2] Mannhold, R., & Van de Waterbeemd, H. (2001). Substructure and whole molecule approaches for calculating log P. *Journal of Computer-Aided Molecular Design*, *15*(4), 337-354.
